# Diurnal Patterns of Energy Intake Derived via Principal Component Analysis and Their Relationship with Adiposity Measures in Adolescents: Results from the National Diet and Nutrition Survey RP (2008–2012)

**DOI:** 10.3390/nu11020422

**Published:** 2019-02-17

**Authors:** Luigi Palla, Suzana Almoosawi

**Affiliations:** 1 Department of Medical Statistics, London School of Hygiene and Tropical Medicine, London WC1E 7HT, UK; 2 Brain, Performance and Nutrition Research Centre, Northumbria University, Newcastle NE1 8ST, UK; suzana.almoosawi@northumbria.ac.uk

**Keywords:** eating time, chrononutrition, dietary pattern, principal component analysis, adolescents, childhood obesity, BMI, waist circumference, chronotype, circadian rhythm

## Abstract

Mounting evidence points towards the existence of an association between energy intake in the evening and an increased prevalence and risk of being overweight and of obesity. The present study aimed to describe diurnal eating patterns (DEP) in a nationally representative sample of UK adolescents and to relate the derived DEP to anthropometrical measures. Data from four-day food records of adolescents aged 11–18 years participating in the 2008–2012 UK National Diet and Nutrition Survey Rolling Programme (NDNS RP) was utilised. The DEP were derived using a principal component analysis on the correlation matrix. Three orthogonal diurnal patterns were interpretable as (i) a linear contrast (8% of total system variation) between breakfast and an earlier lunch vs. a later lunch, late dinner, and evening/night snack, renamed “phase shift” DEP; (ii) a linear contrast (6.0% of system variation) between midmorning snacks, late lunch, and early dinner vs. breakfast, early and late morning snacks, early lunch, midafternoon snacks, and late dinner, renamed “early eating and grazing” DEP; (iii) a linear contrast (6.0% of system variation) between late main meals vs. early main meals and night snacks which was renamed “early main meals and night snacks vs. late main meals” DEP. After the adjustment for confounders, every 1 unit increase in the “early main meals and night snacks vs. late main meals”’ DEP score was significantly associated with a 0.29 kg/m^2^ and 11.6 mm increase in Body Mass Index (BMI) and waist circumference, respectively. There were no significant associations with the other two main DEPs. In conclusion, adolescents who tended to eat large early main meals and night snacks rather than slightly later main meals without night snacks had higher BMI and waist circumference. Further research is required to explore the determinants of DEP and to explore the impact of the context of eating and socioecological factors in the development of specific DEP.

## 1. Introduction

Mounting evidence suggests a potential association between the timing of energy intake and obesity. Most of this evidence is derived from the field of chronobiology wherein clear variations in circadian rhythms of energy metabolism, appetite hormones, and hunger perception have been described and reviewed [1]. Consistent with this, several epidemiological studies [2,3,4] and a meta-analysis of studies in shift-workers [5] have found associations between evening or nighttime energy intake and obesity. This evidence is further reinforced by experimental studies in adults demonstrating that a shift in energy intake towards earlier in the day leads to weight-loss [6] and promotes weight-loss maintenance [7], whilst evening and nighttime energy intake may potentially act as important target points for dietary interventions; focusing on describing the association between one predefined eating occasion or meal slot (i.e., evening or nighttime) and obesity does not provide a holistic perspective on how other eating occasions or meal slots of potential equal importance may influence adiposity [8,9,10]. Indeed, the former approach often ignores the potential complex associations between the various eating occasions, and intake at one eating occasion may impact intake at others. In this respect, numerous studies have indicated that eating behaviours characterised by increased evening or nighttime energy intake are associated with decreased energy intake at breakfast and a general shift in energy intake towards later in the day [11,12,13]. Such findings imply the need for adopting more comprehensive methods for analysing time-of-day data, moving away from the focus on single eating occasions or time slots towards defining whole diurnal eating patterns (DEP). 

Recent epidemiological studies have described the use of latent class analysis [14] and cluster analysis [15] to describe DEP in the general adult population in Australia and Germany respectively. However, to our knowledge, there are no studies describing current DEP in the UK population and how these may relate to obesity prevalence, particularly in adolescence. Understanding current DEP in this age group is critical considering that most eating habits are formed in adolescence and that obesity in adolescence increases the probability of adult obesity by over 50% [16]. Moreover, adolescence is known to be associated with a shift in circadian phase, delayed self-selected sleep time [17], and social jetlag [18]. The implication of these physiological and behavioural changes on DEP and overall health, however, have not yet been elucidated. 

The present study aims to describe DEP in a nationally representative sample of UK adolescents, and to assess the relationship between DEP and body mass index (BMI) and waist circumference as markers of obesity. Based on the cited literature, we hypothesize that diurnal patterns characterized by energy intake in the late evening/night would be associated to a greater BMI and WC.

## 2. Materials and Methods

### 2.1. Study Population

The study population consisted of a complete-case sample of 1438 adolescents aged 11–18 years who participated in the 2008–2012 National Diet and Nutrition Rolling Programme (NDNS RP) and who completed three or four days of dietary assessment. The NDNS RP is a cross-sectional rolling survey that collects yearly information on all food and drinks consumed from approximately a thousand randomly sampled individuals living in private households across the four regions in the UK [19]. 

Individuals were selected from a random sample of 21,573 addresses from 799 postcode sectors obtained between April 2008 and March 2011 from the Royal Mail’s Postcode Address File. Within each selected address, one household was randomly sampled and, if present, one child was randomly selected to participate. The overall response rate for individuals completing three or four days of dietary records was 56% in Year 1, 57% in Year 2, 53% in Year 3, and 55% in Year 4. Details of the survey methodology have been published previously [19]. Ethical approval for the NDNS RP was obtained from the Oxfordshire Research Ethics Committee [19].

### 2.2. Dietary Assessment

Interviewers visited participants in their home, wherein they placed an unweighted food diary to be completed over four consecutive days by survey members. Survey members were provided with written instructions and asked to record everything they ate and drank over the four days, both at home and outside. To ensure compliance and completeness of recording, follow-up checks were scheduled by the interviewer on the second or third day of recording either in person or over the telephone [19]. Home visits were carried out continuously throughout each year, from February 2008 to August 2012, to ensure that seasonal variations in dietary intake were captured. The diary entries were coded and analysed by trained dietary coders using an in-house dietary assessment system DINO (Data In, Nutrients Out). This system is based on food composition data from the Department of Health’s NDNS Nutrient Databank which contains over 7000 regularly updated food codes [19]. Within each diary, the day was subdivided into 24 time intervals coinciding with the hour, and the average of energy intake across the 3 or 4 diet diary days for each one hour interval was calculated.

### 2.3. Anthropometry

Height and weight were measured using a portable stadiometer and weighing scales by trained fieldworkers. Body Mass Index (BMI) was calculated as weight in kilograms divided by height in square meters. Waist circumference was measured at the midpoint between the iliac crest and the lower rib to the nearest 0.1 centimeter.

### 2.4. Additional Covariates

A Computer Assisted Personal Interview (CAPI) was undertaken during the initial visit administered by trained interviewers. The CAPI collected information on the respondent’s socioeconomic characteristics and ethnicity (white vs. non-white) amongst others. Socioeconomic status was defined based on the National Statistics Socioeconomic Classification (NS-SEC) as (1) managerial and professional occupations, (2) intermediate occupations, (3) small employers and own account workers, (4) lower supervisory and technical occupations, (5) semi-routine and routine occupations, and (6) never worked and long-term unemployed. NS-SEC has been used as the primary indicator of socioeconomic status in adolescents in nutrition surveys in the UK [20]. 

Data on sleep were collected by asking an individual survey member “Over the last seven days, that is since last (seven days), how long did you usually sleep for on week nights? That is, Sunday to Thursday nights?” and “And over the last seven days, how long did you usually sleep for on weekend nights? That is, Friday and Saturday nights?” [21]. If the respondent worked on night shifts during the last one week, the average time slept during the day was recorded. If the pattern of time spent in sleep varied widely, interviewers coded the response as “don’t know”. In Years 1–2, the duration of sleep was recorded into a single variable reflecting the time spent asleep in hours and minutes [21]. In Years 3–4, the sleep duration was recorded into two separate variables, one to reflect the hours spent asleep and the second to reflect minutes. To enable data analyses from all years, a new sleep variable was derived to reflect time spent in sleep in hours and fractions of hours. 

### 2.5. Statistical Analysis

Principal Component Analysis (PCA) on the correlation matrix was used to derive DEPs, whereby the 24 variables represented the average hourly energy intake across UK adolescents. Applying PCA on the correlation matrix is equivalent to performing a covariance matrix analysis on standardised variables. This implies that energy intake at each time slot was given the same weight as other time slots regardless of the amount of energy intake. Such an approach removes the influence of the order of magnitude of the intake, ensuring that the eating behaviour at larger meals does not obscure the behavioural patterns at times in between main meals and at night; the main meals would otherwise overwhelmingly account for the variability in individual behaviour, obscuring more subtle diurnal differences. To describe the relationship between energy/macronutrient intake and DEPs, the survey sample estimates of energy, carbohydrates, fats, and proteins by DEP tertiles were calculated for the main diurnal patterns, overall and by hour of the day. Linear regression accounting for the complex survey design was then used to model the association between the derived main diurnal pattern scores and BMI or waist circumference. To account for singleton units, analyses were conducted using the centered method. Models were adjusted by sex, ethnic group, age, socioeconomic status, and total daily energy intake. Overall, 55, 405, 980, 1242, and 4 survey members had missing data on BMI, waist circumference, sleep duration on weekdays, sleep duration on weekends, and NS-SEC, respectively. These values were assumed to be missing at random and consequently imputed using multiple imputations. Fifty imputed data sets were created and fitted by utilising the “ice” and “mim” packages in Stata (StataCorp LP, College Station, TX, USA) [22]. This resulted in an imputed sample of 1497 survey members. Sensitivity analyses were conducted comparing the coefficients derived from imputed data and the complete case analyses.

After multiple imputation, a sensitivity analysis including sleep duration on weekdays and weekends as potential additional confounders was conducted. To identify the differences in DEP between boys and girls, PCA was conducted separately for boys and girls. An additional sensitivity analysis excluding time slots with energy intake less than 210 kJ (50 kcal) was undertaken in accordance with the definition of eating occasions published previously [8].

## 3. Results

Survey members’ characteristics are shown in Table 1 for the complete case sample. Overall, the sample consisted of a complete cases sample of 1438 adolescents.

**Figure 1:**
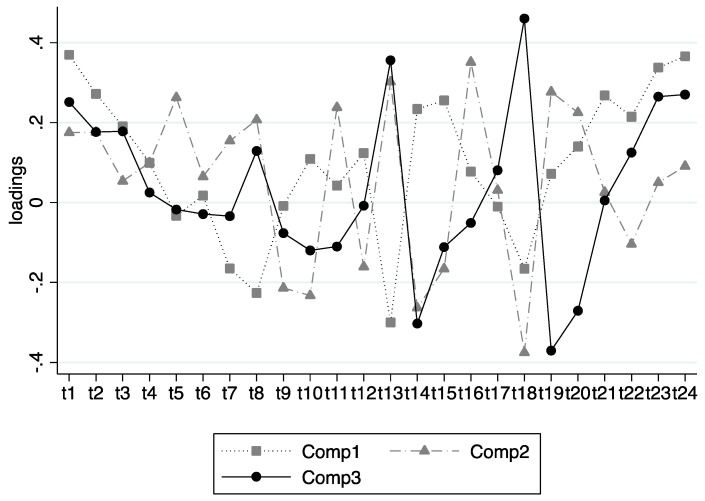
The loadings of the diurnal eating patterns on the variables indicating the energy intake across 24 h of the day based on performing a Principal Components Analysis on the correlation matrix.

Three orthogonal diurnal patterns (Figure 1) were interpretable as (i) a linear contrast (explaining 8% of total system variation) between breakfast and earlier lunch vs. later lunch, late dinner, and evening/night snack which was named “phase shift” DEP (DEP1); (ii) a linear contrast (6.0% of system variation) between midmorning snacks, late lunch, and early dinner vs. breakfast, early and late morning snacks, early lunch, midafternoon snacks, and late dinner, renamed “early eating and grazing” DEP (DEP2); (iii) a linear contrast (6.0% of system variation) between late main meals vs. early main meals and night snacks which was named “antiphase shift” DEP (DEP3). In the univariate analyses, only age and total daily energy intake were significantly associated (positively) with DEP1. For every one-year increment in age, the DEP1 score increased by 0.26 units (*p* < 0.001), indicating that a late/shifted eating pattern to be more pronounced with age. Similarly, for every 100 kcal increase in total daily energy intake, the DEP1 score increase by 0.1 units (*p* < 0.001). Daily energy intake was also associated positively with DEP2, such that every 100 kcal increase in daily energy intake was associated with a 0.3 unit increase in DEP2 (*p* < 0.001), indicating that a shift towards eating late as well as early and frequent eating were related to a higher energy intake. There were no associations between any of the other covariates and remaining dietary patterns. No association between sleep duration on weekdays, sleep duration on weekends, and DEP1–DEP3 were observed; however, the very small size of complete case analysis including them and the likely confounding role of sleep meant we imputed them to be able to include them in the multiple regression models. 

Table 2 presents the estimates of total daily energy and macronutrient intake for each DEP and indicates that the mid-tertile is mostly containing the lowest intake for all DEPs, suggesting a non-linear relationship between DEP and overall intake, particularly for DEP3. There is still a strong evidence of a linear relationship of both DEP1 and DEP2 with all macronutrients/energy (*p*-values < 0.001), which is absent for DEP3. Further detail of tertiles estimated separately by hour of the day are presented in Supplementary Table S4 for DEP1–3.

The association between the derived DEPs and the BMI and waist circumference are shown in Table 3 and Table 4, respectively. In the crude model, only DEP3 was found to be associated positively with BMI and waist circumference. Accordingly, for every 1 unit increase in DEP3, BMI and waist circumference were found to increase by 0.30 kg/m^2^ (95%CI 0.06–0.54; *p* = 0.015) and 1.36 cm (95%CI 0.78–1.93; *p* < 0.001), respectively. After adjusting for sex, ethnicity, age, socioeconomic status, and total mean energy intake over the day, every 1 unit increase in DEP3 was associated with an increase of BMI and waist circumference of 0.28 kg/m^2^ (95%CI 0.05–0.51; *p* = 0.016) and 1.16 cm (95%CI 0.60–1.72; *p* < 0.001), respectively. No association between DEP1 and DEP2 and BMI or waist circumference were observed in either the crude models or the adjusted models. In the complete case analysis, the association was less highly significant in the BMI models where the covariates included in the model appear to have a negligible impact on the effect estimates of DEP3, while in the waist circumference model, each included confounder reduces the effect estimate substantially, although the DEP3 effect remains sizeable and highly significant. In the same analysis including imputed data, there are similar results, with an effect of DEP3 on BMI slightly enhanced in both crude and adjusted analyses (0.31 and 0.29 respectively) and a greater attenuation of the effect of DEP3 on waist circumference (crude = 1.16 and adjusted = 1.03). An additional adjustment by the sleep duration on weekdays and weekends (Supplementary Materials, Table S1) attenuated the DEP3 effect on both measures of adiposity such that every 1 unit increase in DEP3 was associated with an increase in BMI and waist circumference of 0.26 kg/m^2^ (95%CI 0.03–0.49; *p* = 0.026) and 0.99 cm (95%CI 0.44–1.53; *p* < 0.001), respectively.

Additional sensitivity analyses were conducted wherein eating periods were assigned a value of zero if the sum of the energy intake was below 210 KJ (50 kcal). These results are shown in the Supplementary Materials (Figure S4 and Tables S2 and S3). Overall, similar DEPs were derived compared with the initial analyses which did not exclude any eating occasions. The results from the regression analyses using these DEPs did not differ from the original findings. To illustrate, after the adjustment for all covariates, the coefficients for the association between DEP3 and BMI (ß = 0.28, 95%CI 0.03–0.52; *p* = 0.026) or waist circumference (ß = 0.98, 95%CI 0.40–1.57; *p* = 0.001) were somewhat attenuated but remained significant. Furthermore, no interpretative differences in DEPs between boys and girls were observed (Supplementary Materials, Figures S1 and S2). Consequently, DEPs were derived for the full sample. Additional sensitivity analyses applying PCA on the covariance rather than the correlation matrix were conducted. The derived eating patterns are shown in the Supplementary Materials (Figure S3). The latter analysis preserves the order of magnitude of energy intake such that the derived eating patterns give greater weight to eating occasions with larger energy intake and smaller weight to eating occasions with smaller energy intake. Therefore, the resulting eating patterns do not capture the (more subtle) variability of the behaviour of diurnal eating patterns at times with lower energy intake; this is because they mostly reflect the absolute amount of energy intake consumed, which is greater and tends to have greater variance at main meals. Table 2 and Table S4 in the Supplementary Materials confirm that the DEPs we capture in the data by using the correlation matrix are not mainly driven by overall energy intake. 

## 4. Discussion

To our knowledge, this is the first study to describe DEPs in adolescents in the UK using PCA. The study identified three interpretable DEPs in adolescents characterised by different distributions of energy intake across the day. DEP1 was characterised by a phase delay reflecting contrasts between late lunch and nighttime vs. early lunch and breakfast, which we named “phase delay” DEP. DEP2 was reflective of a grazing (frequent eating) eating pattern, and DEP3 reflected contrasts between early lunch and dinner vs. late lunch and dinner. The latter pattern was termed “antiphase” as such a DEP3 could be deemed to be inconsistent with the general tendency of adolescents being late chronotypes [23]. Of the three derived DEPs, only DEP3 was found to be associated positively with BMI and waist circumference in adolescents.

Our findings are consistent in part with the results of a recent analysis of the DEDIPAC-study, which demonstrated that young adults have DEPs characterised by less distinct peaks in the morning and midday and a shift towards meal times later in the day. In the latter study, energy intake in the evening was significantly higher amongst young adults compared to older adults and among overweight men and women compared to normal weight survey members [24]. In the Dortmund Nutritional Anthropometric Longitudinally Designed (DONALD) study, morning energy intake was lower while evening energy intake was higher specifically among adolescents with an evening chronotype. Such phase-shift or delay DEP is consistent with the DEP1 observed in our study, although in the current population, we did not have data on chronotype to test the hypothesis whether DEP1 was more characteristic of adolescents with an evening chronotype. Similarly, Leech and colleagues [14] recently identified through latent class analysis three patterns in Australian adults: conventional, late meal, and grazing which were more recently also found in British adults by the same statistical technique. In the current sample of UK adolescents and using a different, purely descriptive statistical technique (PCA), a grazing pattern was expressed by DEP2 while the late meal behavior is captured by both DEP1 and DEP3. 

Multiple non-modifiable and socioenvironmental factors may have contributed to the observed DEPs. A recent systematic review reported that in young adults, a lack of time is the primary reason for meal skipping [25]. Similar observations have been made in Australian adolescents, wherein lack of time and not being hungry in the morning were found to be the most commonly cited reasons for skipping breakfast [26,27]. A misalignment between adolescents’ circadian clock and external social schedules may be an important contributor to the observed delay in morning energy intake. In this present study, no qualitative data is available to explore the potential determinants of DEPs and no data on circadian typology was collected. However, positive associations between age and DEP1 were observed. Furthermore, adolescents from families with intermediate occupations and lower supervisory and technical occupations were more likely to score lower on DEP2 (tendency to have late breakfast and lunch and early dinner rather than early and frequent eating), compared with adolescents from higher managerial socioeconomic status. 

Mounting evidence suggests that breakfast skipping and increased energy intake in the evening is individually related to increased prevalence of being overweight and of obesity in epidemiological studies [28,29]. However, findings from randomised controlled trials appear to be conflicting [30]. This discrepancy between the epidemiological studies and clinical observations could be ascribed to the fact that few observational studies have investigated the potential intercorrelations between energy intake at various eating occasions or time slots and whether the observed eating behaviours form part of a broader DEP, such that the skipping of breakfast may be associated with greater energy intake later in the evenings. In the UK, one previous study has likewise reported an absence of an association between energy intake after 8 pm and BMI in UK adolescents [31]. However, such an analysis used a predefined cutoff to define the timing of energy intake which may not be relevant to a particular population and ignores the potential complex association between the various eating occasions. Such an average complex pattern of association in individual behaviour could be captured in this current study which identified DEPs with varying eating behaviour at early evening, evening, and nighttime. Previous research has also highlighted that despite evening intake being one of the major contributors to energy intake across the day, variations in the quality of the evening meal exist, which are, in turn, related to differing diet quality over the day [32]. In this present study, each of the derived main DEPs had a component representing energy intake in the evening or nighttime. However, the overall DEPs differed in the contrasts characterising the DEPs and the pattern of energy distribution across the remaining eating occasions during the day. Only DEP3, which was characterised by early main meals combined with nighttime energy intake, was found to be associated with BMI and waist circumference. This finding adds to the current body of literature, since the current standing hypothesis is that a shift in energy intake towards late in the day is associated with an increased total energy intake over the day [33] and adverse health outcomes [28]. In this present study, both DEP1 and DEP2 were associated with increased energy intake over the day. Nonetheless, DEP1 which could be described as a phase delay DEP characterised by breakfast skipping and a shift in energy intake to later in the day, was not related to BMI or waist circumference. Adolescence is generally known to be associated with a phase-delay with the prevalence of evening chronotype being higher in this population subgroup compared to younger children or adults [28]. Accordingly, it could be speculated that the DEP1 may be reflective of the phase delay generally observed in adolescents. The latter is consistent with the observations made by Rossbach and colleagues who found that in adolescents, chronotype is associated with the timing of energy intake, such that a later chronotype is related to a shift in energy intake towards later in the day [11]. More specifically, Rossbach and colleagues found that every 1 h delay in chronotype is associated with a higher prevalence of breakfast skipping and energy intake in the evening [11]. On the balance of this evidence, it could be further hypothesised that, despite adolescents reporting later energy intake through the day, this DEP may not be detrimental to their health since such DEP aligns with the circadian phase shift generally observed in adolescents [11,23]. It could, thus, be deemed that a shift in energy intake later in the day is in-phase with their internal clock [11]. In contrast, DEP3 may reflect the DEP of adolescents who are constrained by social schedules and who, as a result, exhibit a more structured eating pattern characterised by an earlier lunch and dinner. These constraints to comply with socially defined meal times is then counterbalanced by late evening and nighttime energy intake consistent with the circadian phase delay observed in adolescents. It could be speculated that in adolescents, the DEP3 may reflect a behavioural rhythm that is not synchronised with adolescents’ internal circadian phases. Such a misalignment between the biological clock and the behavioural phase may then explain the observed positive association between DEP3 and BMI or waist circumference. Although the potential underlying mechanisms for this association remain to be elucidated, such a hypothesis warrants further investigation. Recently, Munoz and colleagues reported similar findings wherein they found that individuals who ate food at times in-phase with their chronotype regardless of their chronotype were normal weight, whereas overweight or obese individuals reported food intake that was dissociated from their chronotype [34]. 

There are a number of strengths to this study including the national representativeness of the current sample as well as the use of PCA on the correlation matrix to describe the DEP of individuals. Such a PCA reflects diurnal or chronobiological eating patterns rather than eating patterns dependent on the quantity of energy intake as obtained by a PCA on the covariance matrix derived on non-standardised variables.

Previous research often utilised the mean population intake across meal slots as a reflection of the DEP of individuals [10,28], which has been highlighted as a limitation of current epidemiological research in the field of chrononutrition [9]. A main limitation of the current analysis is the assumption that underreporting is equally distributed across eating occasions. Previous research has demonstrated that underreporting is more likely to occur in between main meals [35] and that technology-based dietary assessment methods are more likely to permit the capturing of in-between-meal eating occasions [36]. However, to our knowledge, no methods have been developed to account for such selective misreporting of energy in surveys utilising traditional paper-based dietary assessment methods. A final dietary assessment limitation and one that warrants further attention is related to the dietary sources of energy intake. This is because this current study derived DEPs based on the energy intake occurred within the individual hours of the day. However, we did not account for the dietary sources of energy within such hours to derive the patterns or the differences in the timing of food (or food group) intake, which was outside the methodological scope of this present study. The latter is, however, important considering that various food groups contribute to energy intake at different times of the day.

Other methodological limitations are (i) the limited interpretability of the diurnal pattern scores in that such dimensionally reduced exposures are sample-dependent and convey a qualitative rather than an objective quantitative interpretation that may be used for devising measurable public health interventions and (ii) the use of energy intake data averaged across 3/4 days of diet diary data records rather than using the individual day records. Some information about individual dietary habits may be lost while averaging; further research is warranted to derive both patterns that may be applied easily for public health purposes and able to account for repeated dietary measures. 

Furthermore, the cross-sectional nature of the study design prevents us from ascertaining the direction of causality of the associations; for example, it would be possible that an increase in BMI/WC is itself causing an antiphase shift in adolescents. Randomized trials or prospective longitudinal studies are warranted to confirm the relevance of our findings for public health policies. Assuming the antiphase shift causes BMI/WC, our findings are robust when accounting for the most important confounders, except puberty status as this was not measured in NDNS. 

Although not investigated as part of the current study, previous studies in adults have demonstrated that DEP were associated with differences in diet quality as assessed using the Healthy Eating Index-2005 [15]. Similar observations were made by other studies assessing the relationship between chronotype and food choice. Understanding DEP and how it relates to overall diet quality and food choices may be critical towards guiding the development of eating occasion-specific dietary guidelines, as recently highlighted by the work of Fayet-Moore and colleagues [10]. In the latter study, key opportunities to promote breakfast consumption and to improve food choices at lunch, dinner, and in-between meals eating occasions were identified and described. 

In conclusion, the present study identified three DEPs in a nationally representative sample of adolescents in UK. Adolescents who tended to eat early main meals combined with nighttime energy intake rather than slightly later main meals without nighttime energy intake had higher BMI and waist circumference. Findings from the present study provide evidence to inform public health strategies and dietary guidelines regarding potential eating occasions that may warrant further attention. Further substantive and methodological research is required to explore the determinants of DEP and explore the impacts of the context of eating and socioecological factors in the development of specific DEP.

## Figures and Tables

**Table 1:** The baseline descriptive characteristics (mean (SD) or count/percentage) before multiple imputation for the unweighted survey whole sample and stratified by sex.

Variable	Level	All Sample	Boys	Girls	*p*-Value *
*N*		1497	744	753	
BMI (kg/m^2^)		21.9 (4.3)	21.4 (4.1)	22.4 (4.6)	
Waist circumference (cm)		76.9 (11.1)	78.4 (11.1)	75.4 (10.8)	
DEP1^#^		0.0 (1.4)	0.1 (1.6)	−0.1 (1.3)	0.273
DEP2^#^		0.0 (1.2)	0.1 (1.3)	−0.1 (1.1)	0.114
DEP3^#^		0.0 (1.2)	0.1 (1.3)	−0.1 (1.0)	0.238
Age (years)		14.6 (2.2)	14.5 (2.3)	14.7 (2.1)	0.366
Ethnicity	White	1370 (91.5%)	680 (91.4%)	690 (91.6%)	
	Non-white	127 (8.5%)	64 (8.6%)	63 (8.4%)	0.74
Socioeconomic Status	Q1	597 (40.0%)	288 (38.8%)	309 (41.2%)	
	Q2	122 (8.2%)	67 (9.0%)	55 (7.3%)	0.39
	Q3	167 (11.2%)	806 (10.8%)	87 (11.6%)	
	Q4	149 (10%)	73 (9.8%)	76 (10.1%)	
	Q5	425 (28.5%)	219 (29.5%)	206 (27.5%)	
	Q6	33 (2.2%)	16 (2.2%)	17 (2.3%)	
Total Daily Energy (kcal)		1768.7 (514.5)	1971.4 (527.3)	1568.5 (413.5)	
Sleep duration in weekdays (hours)		8.10 (1.36)	8.11 (1.36)	8.10 (1.37)	0.925
Sleep duration in weekends (hours)		8.66 (1.85)	8.76 (1.65)	8.56 (2.04)	0.671

* *p*-values for the difference between boys and girls: the *p*-values are derived taking into consideration the complex survey design (weights, clusters, and strata). Singleton units (single cluster within a stratum) were centred to enable the estimation of standard errors based on their distance from the grand mean. ^#^ DEP1 is the First Principal Component score (phase shift diurnal eating pattern); DEP2 is the Second Principal Component (early eating and grazing diurnal eating pattern); DEP3 is the Third Principal Component (early main meals and night snacks or antiphase shift diurnal eating pattern).

**Table 2:** The tertile estimates and 95% confidence intervals by DEP for total daily energy and carbohydrate, fat, and protein intake. The *p*-values are calculated based on the linear association with the respective continuous DEP score.

Nutrient	Eating Pattern	Low Tertile	95% CI	Mid Tertile	95% CI	High Tertile	95% CI	*p*-Value
Total Daily Energy	DEP1	1687	(1641, 1733)	1647	(1595, 1700)	1987	(1917, 2057)	<0.001
	DEP2	1779	(1716, 1841)	1654	(1591, 1717)	1889	(1832, 1946)	<0.001
	DEP3	1803	(1742, 1865)	1664	(1602, 1726)	1858	(1799, 1917)	0.252
Total Daily CH	DEP1	231	(224, 237)	222	(215, 229)	263	(253, 272)	<0.001
	DEP2	237	(229, 245)	224	(215, 233)	254	(246, 262)	<0.001
	DEP3	243	(243, 252)	224	(217, 232)	249	(240, 257)	0.748
Total Daily Fat	DEP1	63	(61, 65)	63	(60, 65)	75	(72, 78)	<0.001
	DEP2	69	(65, 72)	61	(59, 64)	71	(69, 74)	0.036
	DEP3	69	(67, 72)	63	(60, 66)	69	(67, 72)	0.809
Total Daily Protein	DEP1	64	(62, 66)	60	(58, 62)	72	(68, 75)	<0.001
	DEP2	66	(63, 68)	61	(59, 64)	69	(66, 71)	0.011
	DEP3	66	(63, 68)	62	(60, 65)	68	(65, 70)	0.324

**Table 3:** The coefficient estimates from the crude and adjusted complete case (a) and imputed (b) multiple regression models relating diurnal eating patterns (exposures) and BMI (outcome), accounting for the complex survey design.

Model	Variable		Coefficient	Lower	Upper	*p*-Value
(a) Crude	DEP1		0.231	−0.019	0.481	0.070
	DEP2		−0.104	−0.327	0.118	0.357
	DEP3		0.299	0.058	0.540	0.015
	Total Energy Intake		−0.001	−0.002	−0.001	<0.001
	Intercept		23.714	22.538	24.891	<0.001
(a) Adjusted	DEP1		−0.145	−0.417	0.126	0.293
	DEP2		−0.032	−0.260	0.195	0.779
	DEP3		0.284	0.054	0.515	0.016
	Sex	girls vs. boys	0.982	0.403	1.560	0.001
	Ethnicity	non-white vs. white	−0.638	−1.637	0.361	0.210
		Q1 (Reference)	-	-	-	-
	Socioeconomic Status	Q2	0.518	−0.411	1.446	0.274
		Q3	1.017	0.073	1.960	0.035
		Q4	1.046	0.071	2.022	0.036
		Q5	0.718	0.054	1.382	0.034
		Q6	1.146	−0.453	2.745	0.159
	Age	years	0.554	0.422	0.685	<0.001
	Total Energy Intake		−0.001	−0.001	0.000	0.043
	Intercept		13.997	11.609	16.386	<0.001
(b) Crude	DEP1		0.237	−0.005	0.479	0.055
	DEP2		−0.068	−0.286	0.150	0.541
	DEP3		0.312	0.080	0.544	0.008
	Total Energy Intake		−0.001	−0.002	−0.001	<0.001
	Intercept		23.801	22.651	24.952	<0.001
(b) Adjusted	DEP1		−0.127	−0.389	0.135	0.340
	DEP2		0.003	−0.218	0.223	0.981
	DEP3		0.291	0.070	0.513	0.010
	Sex	girls vs. boys	1.030	0.459	1.601	<0.001
	Ethnicity	non-white vs. white	−0.708	−1.693	0.277	0.158
		Q1 (Reference)	-	.	.	.
	Socioeconomic Status	Q2	0.554	−0.373	1.481	0.240
		Q3	0.962	0.030	1.893	0.043
		Q4	1.093	0.142	2.043	0.024
		Q5	0.737	0.082	1.391	0.027
		Q6	1.222	−0.652	3.095	0.200
	Age	years	0.543	0.412	0.675	<0.001
	Total Energy Intake		−0.001	−0.001	0.000	0.028
	Intercept		14.217	11.863	16.570	<0.001

**Table 4:** The coefficient estimates from the complete case (a) and imputed (b) crude and adjusted multiple regression models relating diurnal eating patterns (exposures) and waist circumference (outcome), accounting for complex survey design.

Model	Variable		Coefficient	Lower	Upper	*p*-Value
(a) Crude	DEP1		0.473	−0.194	1.140	0.164
	DEP2		−0.224	−0.827	0.378	0.464
	DEP3		1.357	0.782	1.931	<0.001
	Total Energy Intake		0.001	−0.001	0.003	0.318
	Intercept		74.328	71.005	77.650	<0.001
(a) Adjusted	DEP1		−0.310	−0.997	0.377	0.375
	DEP2		0.029	−0.613	0.670	0.930
	DEP3		1.159	0.596	1.722	<0.001
	Sex	girls vs. boys	−2.380	−4.027	−0.733	0.005
	Ethnicity	non-white vs. white	−2.758	−5.628	0.112	0.060
		Q1 (Reference)	-	-	-	-
	Socioeconomic Status	Q2	1.709	−1.160	4.579	0.242
		Q3	2.450	−0.360	5.259	0.087
		Q4	3.762	0.797	6.727	0.013
		Q5	2.053	0.015	4.091	0.048
		Q6	4.435	−0.165	9.035	0.059
	Age	years	1.414	1.018	1.809	<0.001
	Total Energy Intake		0.000	−0.002	0.002	0.869
	Intercept		55.201	47.816	62.585	<0.001
(b) Crude	DEP1		0.604	−0.005	1.214	0.052
	DEP2		−0.227	−0.802	0.349	0.439
	DEP3		1.161	0.603	1.720	<0.001
	Total Energy Intake		0.000	−0.001	0.002	0.722
	Intercept		75.661	72.670	78.651	<0.001
(b) Adjusted	DEP1		−0.448	−0.730	0.525	0.748
	DEP2		−0.011	−0.605	0.583	0.970
	DEP3		1.027	0.496	1.559	<0.001
	Sex	girls vs. boys	−2.364	−3.901	−0.828	0.003
	Ethnicity	non-white vs. white	−2.575	−5.105	−0.045	0.046
		Q1 (Reference)	-	-	-	-
	Socioeconomic Status	Q2	2.100	−0.448	4.647	0.106
		Q3	2.190	−0.333	4.714	0.089
		Q4	2.840	0.245	5.435	0.032
		Q5	2.204	0.469	3.939	0.013
		Q6	4.625	−0.560	9.810	0.080
	Age	years	1.265	0.900	1.629	<0.001
	Total Energy Intake		−0.001	−0.002	0.001	0.423
	Intercept		59.088	52.452	65.723	<0.001

## Supplementary Materials

The following are available online at http://www.mdpi.com/2072-6643/11/2/422/s1, Figure S1: The diurnal eating patterns in boys: The loadings of the diurnal eating patterns on the variables indicate the energy intake across 24 h of the day based on performing a Principal Component Analysis on the correlation matrix (boys only), Figure S2: The diurnal eating patterns in girls: The loadings of the diurnal eating patterns on the variables indicate the energy intake across 24 h of the day, based on performing a Principal Components Analysis on the correlation matrix (girls only), Figure S3: The diurnal eating patterns excluding the eating occasions with <210KJ (50kcal): The loadings of the diurnal eating patterns on the variables indicate the energy intake across 24 h of the day, based on performing a Principal Components Analysis on the correlation matrix, excluding the eating occasions of less than 50 Kcal, Figure S4: The diurnal eating patterns when the Principal Component Analysis is applied on the covariance matrix. The loadings of the diurnal eating patterns on the variables indicate the energy intake across 24 h of the day, based on performing a Principal Components Analysis on the covariance matrix, Table S1: The coefficient estimates for the adjusted imputed multiple regression models additionally including the sleep duration on weekdays and weekends as confounders of the relationship between the diurnal eating patterns (exposures) and BMI (**a**) and Waist Circumference (**b**), accounting for the complex survey design, Table S2: The coefficient estimates from the crude and adjusted complete case regression models relating diurnal eating patterns (exposures) and BMI (outcome), accounting for the complex survey design after excluding eating occasions with energy intake less than 50 kcal, Table S3: The coefficient estimates from the crude and adjusted complete case regression models relating diurnal eating patterns (exposures) and Waist Circumference (outcome), accounting for the complex survey design after excluding eating occasions with energy intake less than 50 kcal, Table S4: The mean and standard error (SE) of energy and macronutrient intake across tertiles of (a) DEP1, (b) DEP2, and (c) DEP3.
