# Supplementary material for: Diurnal Patterns of Energy Intake Derived via Principal Component Analysis and Their Relationship with Adiposity Measures in Adolescents: Results from the National Diet and Nutrition Survey RP (2008–2012)
Source: Nutrients. 2019 Feb 17;11(2):422. doi: 10.3390/nu11020422 (PMC6412640; doi:10.3390/nu11020422)
Supplement: Supplementary file 1 [file nutrients-11-00422-s001.pdf]

SUPPLEMENTARY MATERIALS (Luigi Palla and Suzana Almoosawi)

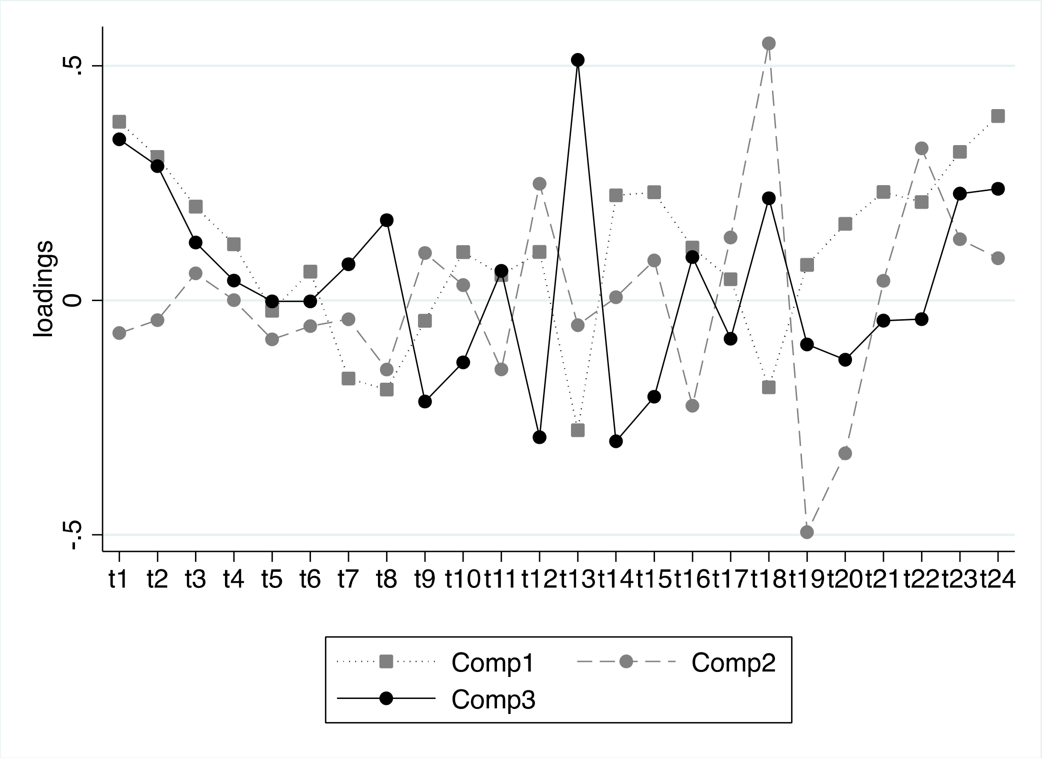

Figure S1 Diurnal eating patterns in boys. Loadings of diurnal eating patterns on the variables indicating energy intake across 24 hours of the day, based on performing Principal Components Analysis on the correlation matrix (boys only)

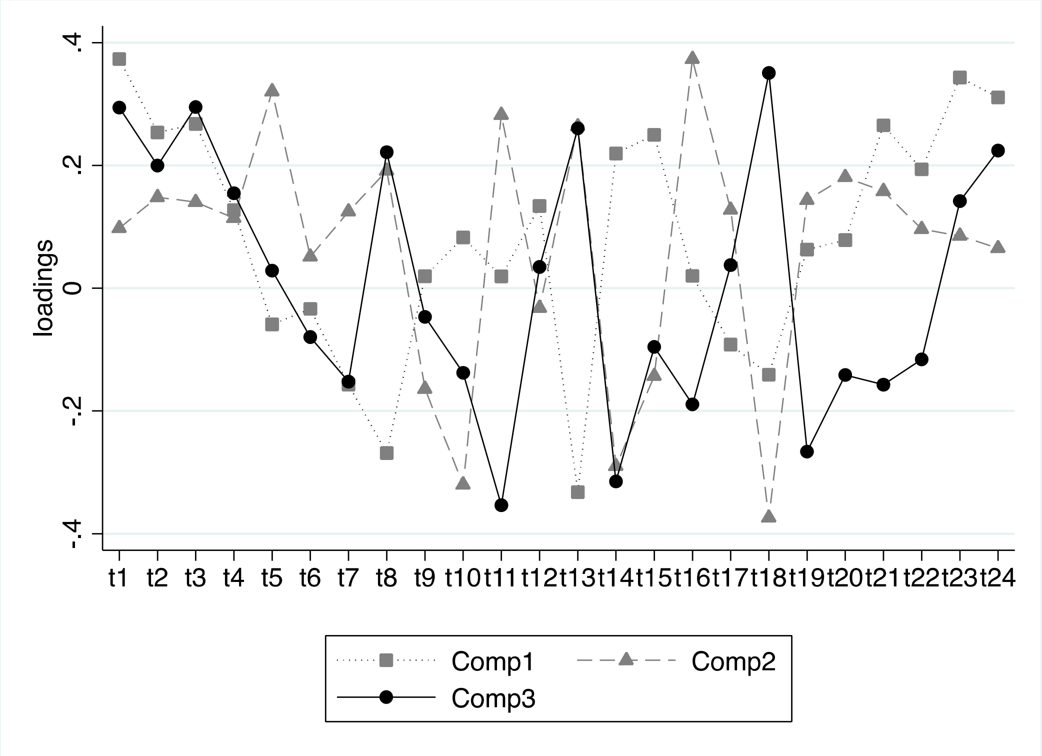

Figure S2 Diurnal eating patterns in girls. Loadings of diurnal eating patterns on the variables indicating energy intake across 24 hours of the day, based on performing Principal Components Analysis on the correlation matrix (girls only).

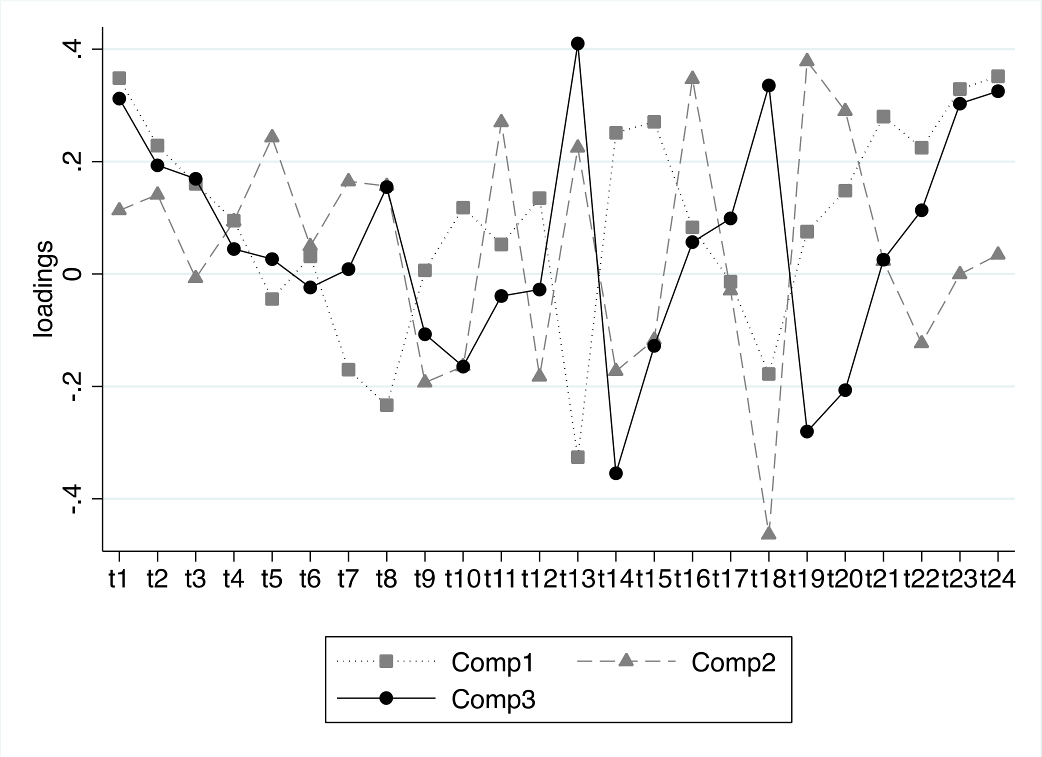

Figure S3 Diurnal eating patterns excluding eating occasions with < 210KJ or 50kcal. Loadings of diurnal eating patterns on the variables indicating energy intake across 24 hours of the day, based on performing Principal Components Analysis on the correlation matrix, excluding eating occasions of less than 50 Kcal

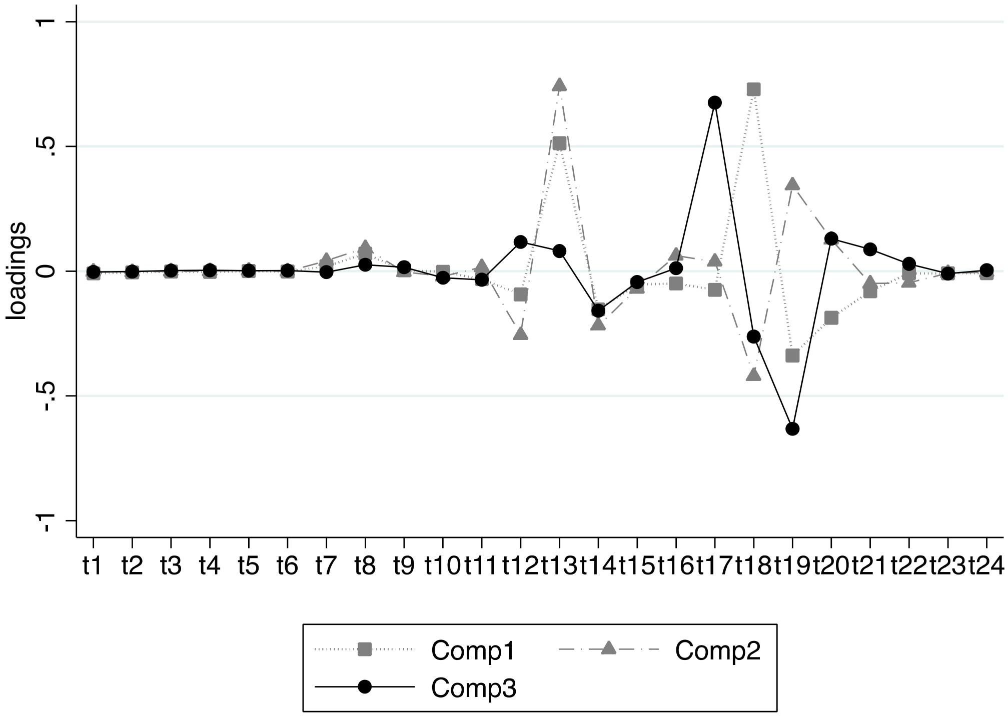

Figure S4 Diurnal eating patterns when Principal Component Analysis is applied on the covariance matrix. Loadings of diurnal eating patterns on the variables indicating energy intake across 24 hours of the day, based on performing Principal Components Analysis on the covariance matrix

Table S1 Coefficient estimates for the Adjusted imputed multiple regression models additionally including sleep duration at weekday and weekends as confounders of the relationship between diurnal eating patterns (exposures) and BMI (a) and Waist Circumference (b), accounting for complex survey design

			Coefficient	Lower	Upper	P-value
(a)BMI	DEP1		-0.104	-0.376	0.167	0.451
	DEP2		-0.011	-0.234	0.212	0.923
	DEP3		0.260	0.031	0.489	0.026
	Sex	girls vs boys	1.061	0.482	1.641	<0.001
	Ethnicity	non-white vs white	-0.762	-1.765	0.241	0.136
		Q1 (Reference)	0.000	.	.	.
	Socioeconomic Status	Q2	0.584	-0.370	1.537	0.229
		Q3	0.916	-0.026	1.858	0.057
		Q4	1.058	0.086	2.029	0.033
		Q5	0.740	0.078	1.403	0.029
		Q6	1.183	-0.703	3.068	0.217
	Age	years	0.535	0.391	0.679	<0.001
	Total Energy Intake		-0.001	-0.001	0.000	0.039
	Sleep at weekdays		-0.204	-0.531	0.123	0.217
	Sleep at weekends		0.094	-0.224	0.411	0.554
	Intercept		15.116	11.018	19.214	<0.001
(b)Waist C.	DEP1		-0.129	-0.793	0.535	0.703
	DEP2		-0.049	-0.654	0.556	0.873
	DEP3		0.986	0.442	1.530	<0.001
	Sex	girls vs boys	-2.429	-3.986	-0.872	0.002
	Ethnicity	non-white vs white	-2.466	-5.073	0.140	0.064
		Q1 (Reference)	0.000	.	.	.
	Socioeconomic Status	Q2	2.200	-0.403	4.803	0.097
		Q3	2.221	-0.339	4.781	0.089
		Q4	2.930	0.297	5.564	0.029
		Q5	2.268	0.516	4.020	0.011
		Q6	4.323	-0.915	9.561	0.105
	Age	years	1.238	0.846	1.629	<0.001
	Total Energy Intake		-0.001	-0.002	0.001	0.470
	Sleep at weekdays		-0.369	-1.203	0.464	0.379
	Sleep at weekends		-0.202	-0.965	0.560	0.596
	Intercept		64.215	52.880	75.550	<0.001

Table S2 Coefficient estimates from the Crude and Adjusted, complete case (a) regression models relating diurnal eating patterns (exposures) and BMI (outcome), accounting for complex survey design after excluding eating occasions with energy intake less than 50 kcal.

			Coefficient	Lower	Upper	P-value
Crude	DEP1		0.215	-0.034	0.465	0.090
	DEP2		-0.122	-0.335	0.091	0.261
	DEP3		0.305	0.058	0.552	0.016
	Total Energy Intake		-0.001	-0.002	-0.001	<0.001
	Intercept		23.712	22.606	24.818	<0.001
Adjusted	DEP1		-0.159	-0.424	0.107	0.241
	DEP2		-0.071	-0.282	0.141	0.511
	DEP3		0.278	0.033	0.524	0.026
	Sex	girls vs boys	1.034	0.460	1.608	<0.001
	Ethnicity	non-white vs white	-0.694	-1.681	0.292	0.167
		Q1 (Reference)	0.000	.	.	.
	Socioeconomic Status	Q2	0.562	-0.363	1.487	0.232
		Q3	0.967	0.034	1.901	0.042
		Q4	1.101	0.150	2.051	0.023
		Q5	0.746	0.091	1.401	0.026
		Q6	1.229	-0.643	3.100	0.197
	Age	years	0.547	0.416	0.677	<0.001
	Total Energy Intake		-0.001	-0.001	0.000	0.038
	Intercept		14.043	11.717	16.369	<0.001

Table S3 Coefficient estimates from the Crude and Adjusted, complete case (a) regression models relating diurnal eating patterns (exposures) and Waist Circumference (outcome), accounting for complex survey design after excluding eating occasions with energy intake less than 50 kcal.

			Coefficient	Lower	Upper	P-value
Crude	DEP1		0.555	-0.077	1.186	0.085
	DEP2		-0.472	-1.037	0.093	0.101
	DEP3		1.116	0.524	1.707	<0.001
	Total Energy Intake		0.000	-0.001	0.002	0.737
	Intercept		75.702	72.850	78.555	<0.001
Adjusted	DEP1		-0.184	-0.827	0.458	0.573
	DEP2		-0.279	-0.856	0.298	0.342
	DEP3		0.984	0.404	1.565	0.001
	Sex	girls vs boys	-2.340	-3.884	-0.795	0.003
	Ethnicity	non-white vs white	-2.554	-5.090	-0.018	0.048
		Q1 (Reference)	0.000	.	.	.
	Socioeconomic Status	Q2	2.103	-0.447	4.654	0.106
		Q3	2.218	-0.324	4.760	0.087
		Q4	2.847	0.249	5.445	0.032
		Q5	2.206	0.466	3.946	0.013
		Q6	4.698	-0.484	9.879	0.075
	Age	years	1.278	0.914	1.641	<0.001
	Total Energy Intake		-0.001	-0.002	0.001	0.463
	Intercept		58.724	52.159	65.289	<0.001

Table S4 Mean and standard error (SE) of energy and macronutrient intake across tertiles of (a) DEP1, (b) DEP2 and (c) DEP3.

(a) DEP1		Energy (kcal)		Protein (g)		Fat (g)		Carbohydrates (g)	
Hour	Tertile	Mean	SE	Mean	SE	Mean	SE	Mean	SE
01:00	1	0.0	0.0	0.0	0.0	0.0	0.0	0.0	0.0
	2	0.1	0.0	0.0	0.0	0.0	0.0	0.0	0.0
	3	10.5	2.1	0.2	0.1	0.3	0.1	1.3	0.2
02:00	1	0.0	0.0	0.0	0.0	0.0	0.0	0.0	0.0
	2	0.0	0.0	0.0	0.0	0.0	0.0	0.0	0.0
	3	10.4	2.7	0.3	0.1	0.3	0.1	1.4	0.4
03:00	1	0.0	0.0	0.0	0.0	0.0	0.0	0.0	0.0
	2	0.0	0.0	0.0	0.0	0.0	0.0	0.0	0.0
	3	3.1	1.7	0.1	0.1	0.1	0.0	0.4	0.2
04:00	1	0.0	0.0	0.0	0.0	0.0	0.0	0.0	0.0
	2	0.1	0.0	0.0	0.0	0.0	0.0	0.0	0.0
	3	4.3	2.9	0.2	0.1	0.2	0.1	0.4	0.3
05:00	1	0.4	0.3	0.0	0.0	0.0	0.0	0.1	0.0
	2	0.0	0.0	0.0	0.0	0.0	0.0	0.0	0.0
	3	0.2	0.1	0.0	0.0	0.0	0.0	0.0	0.0
06:00	1	0.6	0.3	0.0	0.0	0.0	0.0	0.1	0.1
	2	3.7	1.4	0.1	0.0	0.1	0.0	0.6	0.2
	3	2.7	1.3	0.1	0.0	0.1	0.0	0.4	0.2
07:00	1	58.7	6.0	2.0	0.2	1.5	0.2	10.0	1.0
	2	26.8	3.8	0.8	0.1	0.7	0.1	4.6	0.7
	3	11.7	2.1	0.4	0.1	0.3	0.1	2.1	0.4
08:00	1	130.8	6.9	4.3	0.3	3.8	0.3	21.2	1.1
	2	79.3	5.6	2.6	0.2	2.1	0.2	13.5	1.0
	3	43.5	5.8	1.3	0.2	1.3	0.2	7.2	0.9
09:00	1	47.2	4.5	1.6	0.2	1.6	0.2	7.0	0.7
	2	57.5	5.6	1.8	0.2	1.9	0.2	8.9	0.9
	3	51.4	6.3	1.8	0.3	1.8	0.3	7.4	0.7
10:00	1	32.1	3.3	1.0	0.1	1.1	0.1	4.9	0.5
	2	54.8	4.3	1.7	0.2	2.0	0.2	8.0	0.6
	3	72.8	5.6	2.5	0.2	2.7	0.2	10.2	0.8
11:00	1	74.8	6.3	1.9	0.2	3.1	0.3	10.5	0.9
	2	85.3	7.1	2.5	0.3	3.2	0.3	12.3	0.9
	3	98.5	7.4	3.0	0.2	3.6	0.3	14.4	1.0
12:00	1	79.4	8.4	2.8	0.3	3.0	0.3	11.0	1.2
	2	113.9	9.7	3.9	0.4	4.4	0.4	15.5	1.3
	3	147.4	10.8	5.0	0.5	5.6	0.5	20.5	1.4
13:00	1	384.6	12.0	13.8	0.4	15.1	0.5	51.6	1.8
	2	195.6	9.7	7.0	0.4	7.9	0.4	25.8	1.3
	3	136.9	8.5	4.7	0.4	5.5	0.4	18.0	1.1

14:00	1	40.9	4.6	1.4	0.2	1.6	0.2	5.6	0.6
	2	125.2	7.8	4.5	0.3	4.8	0.3	17.1	1.1
	3	182.2	10.4	6.6	0.5	7.3	0.5	24.0	1.4
15:00	1	32.6	3.4	0.8	0.1	1.1	0.1	5.1	0.6
	2	50.1	4.2	1.5	0.2	1.9	0.2	7.1	0.6
	3	125.6	7.7	3.9	0.3	5.1	0.4	17.0	1.0
16:00	1	82.8	6.3	2.1	0.2	3.1	0.3	12.4	0.9
	2	86.9	6.5	2.6	0.3	3.6	0.3	11.7	0.8
	3	105.8	8.4	3.4	0.4	4.3	0.4	14.3	1.1
17:00	1	133.2	10.3	5.4	0.5	5.2	0.4	17.1	1.3
	2	126.9	9.4	5.0	0.4	5.1	0.4	15.9	1.1
	3	126.9	10.2	4.7	0.5	5.0	0.4	16.9	1.3
18:00	1	278.8	13.1	13.7	0.7	10.9	0.6	33.5	1.6
	2	191.0	10.7	8.5	0.5	7.7	0.5	23.2	1.3
	3	153.3	11.1	7.0	0.5	5.8	0.4	19.2	1.4
19:00	1	155.9	10.5	7.7	0.6	5.8	0.4	19.2	1.3
	2	162.1	10.2	7.3	0.5	6.5	0.5	19.7	1.2
	3	166.6	11.1	7.1	0.5	6.5	0.5	20.5	1.4
20:00	1	92.1	7.3	3.6	0.4	3.5	0.3	12.1	0.9
	2	135.5	9.0	5.6	0.4	5.3	0.4	17.2	1.2
	3	186.8	11.6	7.6	0.6	7.1	0.5	23.2	1.5
21:00	1	42.1	3.5	1.2	0.1	1.5	0.1	6.3	0.5
	2	92.5	6.9	2.9	0.2	3.6	0.3	12.5	0.9
	3	179.8	10.5	6.6	0.5	6.6	0.5	22.8	1.3
22:00	1	17.2	2.2	0.4	0.1	0.6	0.1	2.6	0.3
	2	45.8	4.6	1.3	0.2	1.7	0.2	6.4	0.7
	3	94.7	7.3	3.8	0.4	3.5	0.4	11.9	0.9
23:00	1	3.2	0.6	0.1	0.0	0.1	0.0	0.5	0.1
	2	12.4	2.0	0.3	0.1	0.4	0.1	1.8	0.3
	3	47.9	4.1	1.2	0.1	1.5	0.1	6.3	0.6
00:00	1	0.1	0.1	0.0	0.0	0.0	0.0	0.0	0.0
	2	1.8	0.7	0.0	0.0	0.1	0.0	0.3	0.1
	3	23.8	3.6	0.7	0.2	0.7	0.2	2.7	0.4

(b) DEP2		Energy (kcal)		Protein (g)		Fat (g)		Carbohydrates (g)	
Hour	Tertile	Mean	SE	Mean	SE	Mean	SE	Mean	SE
01:00	1	0.6	0.3	0.0	0.0	0.0	0.0	0.1	0.0
	2	2.4	0.7	0.1	0.0	0.1	0.0	0.3	0.1
	3	7.2	1.9	0.1	0.0	0.2	0.1	0.8	0.2
02:00	1	1.0	0.7	0.0	0.0	0.0	0.0	0.1	0.1
	2	0.7	0.3	0.0	0.0	0.0	0.0	0.1	0.0
	3	8.4	2.5	0.3	0.1	0.3	0.1	1.2	0.3
03:00	1	0.1	0.1	0.0	0.0	0.0	0.0	0.0	0.0

	2	0.7	0.6	0.0	0.0	0.0	0.0	0.1	0.1
	3	2.2	1.6	0.1	0.1	0.1	0.0	0.2	0.1
04:00	1	0.0	0.0	0.0	0.0	0.0	0.0	0.0	0.0
	2	0.0	0.0	0.0	0.0	0.0	0.0	0.0	0.0
	3	4.2	2.8	0.2	0.1	0.2	0.1	0.4	0.3
05:00	1	0.0	0.0	0.0	0.0	0.0	0.0	0.0	0.0
	2	0.0	0.0	0.0	0.0	0.0	0.0	0.0	0.0
	3	0.6	0.3	0.0	0.0	0.0	0.0	0.1	0.0
06:00	1	1.0	0.7	0.0	0.0	0.0	0.0	0.1	0.1
	2	3.6	1.4	0.1	0.0	0.1	0.0	0.6	0.2
	3	2.2	1.0	0.1	0.0	0.0	0.0	0.4	0.2
07:00	1	13.6	2.4	0.4	0.1	0.3	0.1	2.4	0.4
	2	34.0	4.8	1.2	0.2	0.7	0.1	6.1	0.9
	3	48.1	5.1	1.5	0.2	1.4	0.2	7.9	0.9
08:00	1	62.8	6.5	2.0	0.2	1.7	0.2	10.6	1.1
	2	64.2	5.2	2.0	0.2	1.7	0.2	11.0	0.9
	3	124.0	7.1	4.2	0.3	3.7	0.2	19.8	1.2
09:00	1	81.2	7.5	2.8	0.3	2.9	0.4	11.7	0.9
	2	43.4	3.7	1.4	0.1	1.4	0.1	6.8	0.6
	3	34.4	4.0	1.1	0.1	1.2	0.2	5.2	0.6
10:00	1	84.6	6.2	2.7	0.2	3.1	0.3	12.1	0.9
	2	48.6	4.0	1.6	0.1	1.7	0.2	7.1	0.5
	3	29.8	3.0	0.9	0.1	1.1	0.1	4.4	0.4
11:00	1	64.0	6.7	1.8	0.2	2.2	0.3	9.7	1.0
	2	79.3	6.0	2.2	0.2	2.9	0.2	11.8	0.9
	3	112.0	8.3	3.4	0.3	4.6	0.4	15.2	1.0
12:00	1	148.3	12.0	5.2	0.6	5.8	0.6	19.9	1.4
	2	120.1	10.2	4.2	0.4	4.4	0.4	16.8	1.5
	3	76.4	6.9	2.5	0.3	2.9	0.3	10.7	1.0
13:00	1	156.7	9.4	5.4	0.4	6.1	0.4	21.3	1.2
	2	218.1	11.1	7.9	0.4	8.5	0.5	29.3	1.6
	3	335.4	11.9	12.0	0.4	13.5	0.5	44.0	1.7
14:00	1	176.4	9.6	6.3	0.4	7.1	0.4	23.2	1.3
	2	101.0	7.7	3.6	0.3	4.0	0.3	13.6	1.0
	3	76.2	6.9	2.7	0.3	2.9	0.3	10.5	1.0
15:00	1	102.0	7.0	2.9	0.3	4.1	0.3	14.1	1.0
	2	67.8	6.2	2.1	0.2	2.6	0.3	9.6	0.9
	3	42.9	4.3	1.3	0.2	1.6	0.2	6.1	0.6
16:00	1	47.7	5.8	1.3	0.2	1.9	0.2	6.8	0.8
	2	78.7	5.5	2.3	0.2	3.1	0.3	11.0	0.8
	3	143.0	7.8	4.2	0.3	5.7	0.4	19.8	1.1
17:00	1	116.4	8.7	4.7	0.4	4.6	0.4	14.9	1.0
	2	133.4	10.6	5.3	0.5	5.1	0.4	17.5	1.4
	3	136.1	9.5	5.1	0.4	5.5	0.4	17.4	1.2

18:00	1	329.0	13.2	15.6	0.7	13.3	0.6	39.2	1.6
	2	192.0	10.6	9.4	0.6	7.4	0.4	23.3	1.3
	3	118.7	8.0	5.1	0.4	4.5	0.3	15.4	1.0
19:00	1	102.9	7.5	4.4	0.4	4.0	0.3	12.8	0.9
	2	134.1	8.4	6.1	0.5	5.3	0.4	16.4	1.0
	3	238.8	12.2	11.2	0.7	9.2	0.5	29.3	1.5
20:00	1	101.2	7.6	3.8	0.4	3.9	0.3	13.1	1.0
	2	127.7	8.9	5.4	0.4	5.0	0.4	15.9	1.1
	3	179.8	11.6	7.4	0.6	6.9	0.5	22.8	1.5
21:00	1	96.5	8.2	3.4	0.4	3.7	0.4	12.8	1.0
	2	119.9	9.1	4.2	0.4	4.5	0.4	15.6	1.1
	3	97.4	7.4	3.2	0.3	3.5	0.3	13.1	1.0
22:00	1	70.9	8.3	2.3	0.4	2.8	0.4	9.1	1.0
	2	55.4	5.4	1.8	0.2	2.0	0.2	7.5	0.7
	3	33.6	3.8	1.3	0.1	1.1	0.1	4.5	0.5
23:00	1	17.8	2.0	0.5	0.1	0.6	0.1	2.5	0.3
	2	20.0	2.7	0.5	0.1	0.6	0.1	2.8	0.4
	3	25.4	3.3	0.7	0.1	0.8	0.1	3.4	0.5
00:00	1	4.1	1.1	0.1	0.0	0.1	0.1	0.5	0.1
	2	8.7	2.3	0.3	0.1	0.3	0.1	1.0	0.2
	3	12.6	2.8	0.3	0.1	0.4	0.1	1.5	0.3

(c) DEP3		Energy (kcal)		Protein (g)		Fat (g)		Carbohydrates (g)	
Hour	Tertile	Mean	SE	Mean	SE	Mean	SE	Mean	SE
01:00	1	0.3	0.2	0.0	0.0	0.0	0.0	0.0	0.0
	2	0.9	0.5	0.0	0.0	0.0	0.0	0.1	0.1
	3	10.0	2.2	0.2	0.0	0.2	0.1	1.2	0.3
02:00	1	0.4	0.2	0.0	0.0	0.0	0.0	0.1	0.1
	2	1.2	0.5	0.1	0.0	0.0	0.0	0.1	0.1
	3	9.4	2.9	0.2	0.1	0.3	0.1	1.3	0.4
03:00	1	0.0	0.0	0.0	0.0	0.0	0.0	0.0	0.0
	2	0.1	0.0	0.0	0.0	0.0	0.0	0.0	0.0
	3	3.2	1.9	0.2	0.1	0.1	0.1	0.4	0.2
04:00	1	0.0	0.0	0.0	0.0	0.0	0.0	0.0	0.0
	2	2.9	2.8	0.1	0.1	0.1	0.1	0.3	0.3
	3	1.6	1.0	0.1	0.1	0.1	0.0	0.2	0.1
05:00	1	0.3	0.3	0.0	0.0	0.0	0.0	0.0	0.0
	2	0.0	0.0	0.0	0.0	0.0	0.0	0.0	0.0
	3	0.2	0.1	0.0	0.0	0.0	0.0	0.1	0.0
06:00	1	5.0	1.7	0.1	0.0	0.1	0.1	0.9	0.3
	2	0.9	0.4	0.0	0.0	0.0	0.0	0.1	0.1
	3	0.6	0.3	0.0	0.0	0.0	0.0	0.1	0.0
07:00	1	35.0	4.0	1.1	0.1	0.9	0.1	5.9	0.7

	2	35.1	5.1	1.2	0.2	0.8	0.1	6.1	0.9
	3	27.4	4.1	0.8	0.1	0.7	0.1	4.8	0.8
08:00	1	72.0	6.1	2.3	0.2	2.1	0.2	11.6	1.0
	2	74.7	6.3	2.5	0.2	1.9	0.2	12.7	1.1
	3	109.9	8.4	3.6	0.3	3.1	0.3	18.0	1.4
09:00	1	63.0	6.0	2.0	0.2	2.1	0.2	9.5	0.9
	2	51.6	6.4	1.8	0.3	1.8	0.3	7.4	0.7
	3	39.5	3.5	1.3	0.1	1.3	0.2	6.0	0.5
10:00	1	69.1	5.1	2.2	0.2	2.7	0.2	9.7	0.7
	2	50.3	4.0	1.6	0.2	1.7	0.2	7.7	0.6
	3	37.7	4.0	1.3	0.2	1.3	0.2	5.5	0.6
11:00	1	104.4	8.1	3.1	0.3	4.0	0.4	14.8	1.1
	2	76.5	5.4	2.3	0.2	2.9	0.2	11.0	0.7
	3	75.2	7.1	2.0	0.2	2.9	0.3	11.0	1.0
12:00	1	106.2	8.5	3.6	0.3	3.9	0.3	15.0	1.2
	2	114.8	10.6	4.1	0.5	4.4	0.5	15.5	1.3
	3	120.1	10.7	4.1	0.4	4.7	0.4	16.4	1.5
13:00	1	154.8	8.7	5.4	0.3	6.1	0.4	20.7	1.1
	2	234.0	10.4	8.5	0.4	9.3	0.5	30.9	1.3
	3	345.3	14.2	12.3	0.5	13.7	0.6	46.1	2.1
14:00	1	183.4	10.2	6.7	0.5	7.3	0.5	24.3	1.4
	2	91.1	7.0	3.0	0.3	3.6	0.3	12.3	1.0
	3	63.1	6.0	2.3	0.3	2.4	0.2	8.6	0.8
15:00	1	93.0	7.5	3.0	0.3	3.7	0.3	12.7	1.0
	2	57.0	5.0	1.5	0.2	2.2	0.2	8.3	0.7
	3	55.7	4.7	1.6	0.2	2.1	0.2	7.9	0.7
16:00	1	100.1	7.3	2.8	0.3	4.0	0.3	14.1	1.0
	2	101.6	8.1	3.2	0.3	4.1	0.4	13.6	1.0
	3	72.4	5.5	2.0	0.2	2.7	0.3	10.6	0.8
17:00	1	103.4	9.0	3.6	0.4	4.1	0.4	13.8	1.1
	2	134.7	9.2	5.4	0.4	5.4	0.4	16.9	1.2
	3	152.7	12.1	6.3	0.5	6.0	0.5	19.7	1.5
18:00	1	96.8	6.5	4.4	0.3	3.7	0.3	12.1	0.8
	2	201.3	9.6	9.4	0.5	7.9	0.4	24.6	1.1
	3	343.0	13.9	16.3	0.7	13.6	0.6	41.3	1.7
19:00	1	253.1	11.8	11.7	0.6	10.0	0.5	30.7	1.4
	2	136.4	9.4	6.3	0.5	5.2	0.4	17.0	1.2
	3	82.7	7.4	3.6	0.4	3.1	0.3	10.3	0.9
20:00	1	204.0	11.8	8.6	0.6	7.9	0.5	25.7	1.5
	2	116.8	7.7	4.9	0.4	4.5	0.3	14.9	1.0
	3	84.2	7.9	2.9	0.3	3.2	0.3	10.8	1.0
21:00	1	107.9	7.7	3.7	0.3	4.2	0.3	14.2	1.0
	2	106.9	8.6	3.9	0.4	4.1	0.4	14.3	1.0
	3	98.8	8.0	3.2	0.3	3.4	0.3	13.0	1.1

22:00	1	38.0	4.7	1.0	0.2	1.4	0.2	5.2	0.6
	2	55.8	6.9	1.7	0.3	2.2	0.3	7.4	0.8
	3	65.6	6.2	2.8	0.3	2.3	0.3	8.4	0.8
23:00	1	11.6	1.9	0.3	0.1	0.4	0.1	1.7	0.3
	2	17.0	2.2	0.4	0.1	0.5	0.1	2.6	0.3
	3	36.5	3.9	0.9	0.1	1.1	0.1	4.5	0.5
00:00	1	1.3	0.3	0.0	0.0	0.0	0.0	0.2	0.1
	2	2.6	0.6	0.1	0.0	0.1	0.0	0.3	0.1
	3	23.3	3.8	0.7	0.2	0.7	0.2	2.6	0.4
